# A Multivariate Blaschke-Based Mode Decomposition Approach for Gear Fault Diagnosis

**DOI:** 10.3390/s25206302

**Published:** 2025-10-11

**Authors:** Xianbin Zheng, Zhengyang Cheng, Junsheng Cheng, Yu Yang

**Affiliations:** College of Mechanical and Vehicle Engineering, Hunan University, Changsha 410082, China

**Keywords:** Multivariate Blaschke-based Mode Decomposition, stochastic adaptive fourier decomposition, joint spectrum segmentation, gear fault diagnosis, feature extraction

## Abstract

Existing multivariate signal decomposition methods insufficiently account for the mechanical characteristics of gear systems, limiting their capability in fault feature extraction. To address this limitation, we propose a novel method, Multivariate Blaschke-based Mode Decomposition (MBMD). In MBMD, multivariate vibration signals are modeled as multi-dimensional responses of the gear system. Using Stochastic Adaptive Fourier Decomposition (SAFD), these signals are represented as a unified combination of Blaschke products, enabling adaptive multi-channel information fusion. To achieve modal alignment, we introduce the concept of Blaschke multi-spectra, reformulating the decomposition problem as a spectrum segmentation task, which is solved via a joint spectral segmentation algorithm. Furthermore, a voting-based filter bank, designed according to gear fault mechanisms, is employed to suppress noise and enhance fault feature extraction. Experimental validation on gear fault signals demonstrates the effectiveness of MBMD, showing that it can efficiently integrate multivariate information and achieve more accurate fault diagnosis than existing methods, providing a new perspective for mechanical fault diagnosis.

## 1. Introduction

Gears, as essential components of mechanical transmission systems, are widely used across industries such as wind power [1], aviation [2], marine [3], automotive [4], and industrial equipment [5,6]. Their operational condition directly impacts the reliability, efficiency, and safety of the entire system. However, due to prolonged exposure to alternating loads, impact vibrations, and complex working conditions, gears are highly susceptible to failures such as wear, pitting, and chunk missing. Undetected faults can result in equipment failures, production downtime, or even severe safety incidents [7,8]. As a result, gear health monitoring and fault diagnosis have long been a key focus in mechanical condition monitoring.

Traditional gear signal analysis methods primarily rely on single-channel signals [9,10,11]. However, single-channel data have limited dimensionality, are vulnerable to noise interference, and often fail to comprehensively capture the multi-dimensional fault characteristics of gears. In recent years, multi-channel signal decomposition methods have become an important research direction in gear fault diagnosis due to their ability to offer information complementarity, noise resistance, and a more comprehensive representation of fault characteristics [12,13]. The main challenge is effectively fusing multi-channel information and extracting physically meaningful fault features.

Multivariate Empirical Mode Decomposition (MEMD) [14,15] aligns multi-channel signals using Hammersley projection, which requires no predefined basis functions, ensuring that the intrinsic mode functions (IMFs) have consistent physical meaning. However, MEMD suffers from low computational efficiency and inherent mixing issues. To overcome these limitations, Multivariate Local Characteristic-Scale Decomposition (MLCD) [16] enhances computational efficiency by replacing the iterative sifting process used in MEMD with piecewise linear transformations. Subsequent improvements resulted in Completely Adaptive Projection MLCD (CAPMLCD) [17], which adapts projection methods dynamically to address power imbalance in multi-channel signals. However, both MLCD and CAPMLCD are dependent on extremum interpolation, which makes them vulnerable to noise interference.

To address the shortcomings of MEMD, Rehman et al. proposed Multivariate Variational Mode Decomposition (MVMD) [18], which extends the variational optimization framework to multi-channel signal processing. MVMD achieves synchronous separation of multi-modal signals by minimizing the total bandwidth of all channel IMFs. However, its performance is highly dependent on preset parameters (e.g., mode number, penalty factor), leading to instability in decomposition. To improve decomposition accuracy, Liu et al. introduced the Successive Multivariate VMD (SMVMD) [19], which formulates an optimization problem using an instantaneous linear mixing model to achieve more accurate modal separation. Despite these advancements, both MVMD and SVMD assume a certain degree of inter-channel correlation. In real-world applications, however, inter-channel signals may exhibit weak or complex correlations, which limits the effective use of multi-channel information and can lead to inaccurate decomposition results.

In recent years, some researchers have proposed converting complex signal analysis into Blaschke product analysis [20,21]. As an important tool in complex analysis, the Blaschke product can extract specific frequency components or features from a signal, and it has attracted widespread attention in mathematical analysis and signal processing.

Adaptive Fourier Decomposition (AFD) [22] is a well-established signal decomposition technique grounded in the Blaschke product. It dynamically decomposes nonlinear, non-stationary signals into a series of non-negative frequency components, following the principle of maximum selection. However, when handling complex mechanical vibration signals, AFD tends to decompose many meaningless modes, making it difficult to uncover the internal characteristic information of the vibration signal. To overcome the limitations of AFD, Blaschke Mode Decomposition (BMD) [23] was proposed. BMD proposes converting complex signals into Blaschke mono-components based on fault characteristic indicators, and then decomposing the signals into a series of modes with energy concentration tendencies through spectral segmentation, significantly reducing noise interference and extracting mechanical fault feature information. However, like single-channel methods, BMD can only process vibration information from a single direction and is not effective in handling multi-source vibration signals.

Stochastic Adaptive Fourier Decomposition (SAFD) [24,25] is a new sparse representation method that can represent random signals/systems with potential data distributions as a unified Blaschke product form. However, SAFD is only suitable for analysis and cannot extract fault features or eliminate noise interference, making it unsuitable for dealing with strong noise and complex vibration signals.

From the above analysis, it can be seen that single-channel methods can only handle vibration data from a single direction, leading to potential fault information loss. Although existing multi-channel methods can handle multi-dimensional vibration data, they do not fully consider the mechanical system characteristics during the decomposition process, making fault feature extraction difficult.

Inspired by SAFD and BMD, this paper proposes a Multivariate Blaschke-based Mode Decomposition (MBMD) method. MBMD is primarily divided into three steps: First, MBMD treats multivariate vibration signals as the observed responses of a gear system in different dimensions. It employs Stochastic Adaptive Fourier Decomposition (SAFD) to invert the gear system, representing the multivariate vibration signals with a unified Blaschke product, thereby achieving multi-channel information fusion. Second, MBMD proposes Blaschke multi-spectra, transforming the multivariate signal decomposition problem into multivariate spectrum segmentation problem. Based on this, joint spectral segmentation algorithm is proposed to partition the Blaschke multi-spectra into segments with the same Blaschke product, thereby achieving modal alignment. Finally, based on the gear fault mechanism, MBMD designs a voting filter bank to eliminate noise interference in the modes and accurately extract fault feature information. The main contributions of this paper are as follows:

(1) A multivariate signal decomposition method, MBMD, is proposed. MBMD treats multivariate vibration signals as the observed responses of a gear system in different dimensions. It is the first to introduce the SAFD theory into the mechanical fault diagnosis field, inverting the gear system and representing multivariate vibration signals using a unified Blaschke product, thereby achieving multivariate information fusion. Its decomposition mechanism is completely different from existing multivariate signal decomposition methods.
(2) The concept of the Blaschke multi-spectra is proposed, representing multivariate signals in the form of a Blaschke multi-spectra. Building on this, a joint spectral segmentation approach is introduced. This strategy divides the Blaschke multi-spectra into several segments with concentrated energy, enabling the subsequent decomposition results to be organized on the same Blaschke product scale. This lays the groundwork for multivariate decomposition and ensures modal alignment.
(3) A voting filter bank is proposed to extract mechanical fault feature components, accurately eliminating noise interference.

The remaining structure of this paper is as follows: Section 2 elaborates the MBMD theory, Section 3 presents experimental signal analysis, and Section 4 provides the conclusion.

## 2. Multivariate Blaschke-Based Mode Decomposition

### 2.1. SAFD and Blaschke Multi-Spectra

Stochastic Adaptive Fourier Decomposition (SAFD) is an emerging mathematical theory capable of representing stochastic signals with underlying probability distributions in a unified Blaschke product form.

The multivariate signals are vibrations measured at different positions and in different directions of the gear system. In other words, multivariate signals can be regarded as observed responses from different dimensions of the gear system. Conversely, if we could obtain all the observed responses of the gear system, we should theoretically be able to use these responses to approximate the gear system. We propose simplifying the gear system by viewing the gear vibration system as a collection composed of countless observed responses, as expressed in Equation (1).

(1) $$F(t,c)=\{f_{1}(t),f_{2}(t),\ldots ,f_{C}(t)\}$$

where F(t,c) is the simplify gear system, $\left\{f_{1},f_{2},\ldots ,f_{C}\right\}$ is multivariate signals of gear system, c is the different dimensional of system, ∀c∈[1,C], and t is time variables.

Since the multi-channel gear vibration signals are governed by the same vibration source, they can be considered as stochastic signals sharing an identical underlying distribution.

According to SAFD theory, the multivariate signal $\left\{f_{1},f_{2},\ldots ,f_{C}\right\}$ can be represented by a common set of weighted Blaschke products, with weighted Blaschke product expressed in Equation (2).

(2) $$B_{n}=\frac{\sqrt{1-{\left|a_{n}\right|}^{2}z}}{1-{\bar{a}}_{n}z}\prod_{k=1}^{n}\frac{z-a_{k}}{1-{\bar{a}}_{k}z}$$

where $B_{n}$ represents the weighted Blaschke product, and $z=e^{it}$, $\left\{a_{1},a_{2},\ldots ,a_{k}\right\}$ denotes the control parameter of $B_{n}$.

At this point, F(t,c) can be reconstructed according to Equation (3).

(3) $$f_{c}(t)=\sum_{n=1}^{\infty }\langle f_{c},B_{n}\rangle B_{n}$$

where $\langle \cdot \rangle$ denotes the inner product operator.

The weighted Blaschke product is governed by a set of parameters $\left\{a_{1},a_{2},\ldots ,a_{n}\right\}$, which can be determined through the stochastic maximal selection principle as shown in Equation (4).

(4) $$a_{k}=\arg\max\{E_{c}\left|\langle f_{c},B_{a_{k}}\rangle \right|\}$$

where $E_{c}$ represents the mathematical expectation under the condition c.

For the F(t,c), taking $\left\{B_{1},B_{2},\ldots ,B_{n}\right\}$ as the system’s basis functions (where $B_{1}$ is defined as the first-order, $B_{2}$ as the second-order, and so on, with $B_{n}$ as the *n-th* order), $\langle f_{c},B_{n}\rangle$ represents the projection of $f_{c}$ onto the basis function $B_{n}$. Equation (3) can be interpreted as the inversion process of F(t,c) using $\left\{B_{1},B_{2},\ldots ,B_{n}\right\}$.

We represent any response $f_{c}$ using the $S_{c}$, as shown in Equation (5):

(5) Sc=Re{fc,B1,fc,B2,…,fc,Bn}

where Re denotes the complex real-part operator.

Thus, $\left\{f_{1},f_{2},\ldots ,f_{C}\right\}$ can be constructed as a linear combination of the common basis $\left\{B_{1},B_{2},\ldots ,B_{n}\right\}$, and Blaschke multi-spectra is subsequently derived based on $\left\{B_{1},B_{2},\ldots ,B_{n}\right\}$, as formalized in Equation (6).

(6) S1=Re{f1,B1,f1,B2,…,f1,Bn}S2=Re{f2,B1,f2,B2,…,f2,Bn}⋮SC=Re{fC,B1,fC,B2,…,fC,Bn}

At this point, the decomposition problem of mechanical multivariate vibration signals can be transformed into a segmentation problem of the Blaschke multi-spectra $\left\{S_{1},S_{2},\ldots ,S_{C}\right\}$.

### 2.2. Joint Spectrum Segmentation

To further integrate multivariate signal information, an ideal strategy is to use the shared spectral lines for unified segmentation according to the trend of the Blaschke multi-spectra, so that corresponding modes share the same Blaschke product. We propose a joint spectral segmentation algorithm and provide the following assumptions for joint spectral segmentation:

1. The Blaschke multi-spectra can be divided into ***K*** spectral segments, each sharing the same Blaschke product.
2. There exist ***K + 1*** shared spectral lines, such that the ***K*** segmented spectral segments exhibit a distinct energy concentration trend.

The joint spectral segmentation algorithm takes unimodal intervals as the basic unit, converting the joint spectral segmentation problem into a combinatorial optimization problem. It uses the branch-and-bound method to find the optimal shared spectral lines, thereby achieving modal alignment and multivariate information fusion.

**(a)**  **Unimodal interval**

Assuming that the Blaschke multi-spectra is monotonically increasing in a certain interval $\left[a_{1},c_{1}\right]$ and monotonically decreasing in interval $\left[c_{1},b_{1}\right]$, it can be considered that interval $\left[c_{1},b_{1}\right]$ exhibit a unimodal pattern, with energy predominantly concentrated at the peak $c_{1}$ of the sequence.

If a segment of the Blaschke multi-spectra approximates a unimodal pattern, we can classify this segment as having a statistically significant ‘unimodal characteristic’ referred to as a unimodal interval. The criteria for determining a statistically significant unimodal interval are outlined in references [26,27], and will not be further discussed here. We regard the unimodal interval as the fundamental unit of the Blaschke multi-spectra, and the following details describe its algorithmic implementation.

First, a moving smoothing filter is applied to the Blaschke multi-spectra to obtain a smoothed multivariate spectrum, denoted as $\left\{{\hat{S}}_{1},{\hat{S}}_{2},\ldots ,{\hat{S}}_{C}\right\}$. Then, the local maxima and minima of $\left\{{\hat{S}}_{1},{\hat{S}}_{2},\ldots ,{\hat{S}}_{C}\right\}$ are computed. The adjacent local maxima and minima are used as boundaries for monotonic continuous segments. At this point, the smoothed multivariate spectrum can be represented as a combination of multiple monotonic continuous segments, as shown in Equation (7).

(7) $$\left\{\begin{array}{c} {\hat{S}}_{1}=\{s_{11},s_{12},\ldots ,s_{1k}\}\\ {\hat{S}}_{2}=\{s_{21},s_{22},\ldots ,s_{2k}\}\\ \vdots \\ {\hat{S}}_{C}=\{s_{C1},s_{C2},\ldots ,s_{Ck}\} \end{array}\right.$$

where for ∀j∈[1,k] and ∀i∈[1,C], interval $s_{ij}$ is monotonic, while the monotonicity of adjacent continuous segments may not be consistent.

Choose the ***j-th*** segment of ${\hat{S}}_{i}$, and try to combine the adjacent segments $s_{ij}$ into a unimodal segment $\left[s_{i(j-1)},s_{i(j+1)}\right]$ that meets the statistical criteria for unimodality. ${\hat{S}}_{i}$ will be updated according to Equation (8).

(8) $${\hat{S}}_{i}=\{s_{i1},\ldots ,s_{i(j-1)},s_{i(j+1)},\ldots ,s_{ik}\}$$

The segment merging process is repeated continuously until no further merging is possible in ${\hat{S}}_{i}$. The resulting unimodal segmentation is denoted as:

(9) $${\hat{S}}_{i}=\{{\hat{s}}_{i1},\ldots ,{\hat{s}}_{iL}\}$$

where ${\hat{s}}_{il}$ is unimodal interval in Blaschke multi-spectra.

By applying the above process to all the Blaschke multivariate spectra, the corresponding unimodal intervals of the multivariate spectra can be obtained, as shown in Equation (10).

(10) $$\left\{\begin{array}{c} {\hat{S}}_{1}=\{{\hat{s}}_{11},{\hat{s}}_{12},\ldots ,{\hat{s}}_{1L_{1}}\}\\ \vdots \\ {\hat{S}}_{C}=\{{\hat{s}}_{C1},{\hat{s}}_{C2},\ldots ,{\hat{s}}_{CL_{C}}\} \end{array}\right.$$

where $L_{1},\ldots ,L_{C}$ is the number of unimodal intervals for $\left\{{\hat{S}}_{1},{\hat{S}}_{2},\ldots ,{\hat{S}}_{C}\right\}$.

**(b)**  **Alignment**

As the basic unit of the multivariate spectrum, the unimodal interval exhibits energy concentration. However, the number of unimodal intervals differs across channels, making modal alignment impossible. Modal alignment is an essential property of multivariate signal decomposition, meaning that the N-th mode of all channel signals should correspond to the same Blaschke product. Therefore, it is necessary to construct shared spectral lines to achieve joint spectral segmentation.

Analysis reveals that using the boundaries of unimodal intervals as shared spectral lines ensures that the spectral segments of a channel are composed of a finite number of unimodal intervals. Additionally, some unimodal intervals have lower energy and bandwidth, and their energy concentration characteristics are less pronounced compared to the overall multivariate spectrum.

Thus, we convert the joint spectral segmentation problem into a combinatorial optimization problem, where the goal is to find shared spectral lines from the set of all unimodal interval boundaries, such that the spectral segments exhibit an energy concentration trend and ensure the integrity of the main feature components. (The core idea is to preserve the integrity of important unimodal intervals while splitting secondary unimodal intervals when necessary.)

The mathematical definition of the joint spectral segmentation problem is as follows: Given Blaschke multivariate spectra $\left\{S_{1},S_{2},\ldots ,S_{C}\right\}$ formed by $\left\{B_{1},B_{2},\ldots ,B_{n}\right\}$, the goal is to find ***K + 1*** shared spectral lines $\left\{b_{1},\ldots ,b_{K+1}\right\}$ (where $1=b_{1}<b_{2}<\ldots <b_{K+1}=n$) that divide the $\left\{S_{1},S_{2},\ldots ,S_{C}\right\}$ into C×K subintervals, such that all spectral segments exhibit energy concentration.

The objective function for joint spectral segmentation is shown in Equation (11).

(11) $$\underset{\left\{b_{1},\ldots ,b_{K+1}\right\}}{\max}f=\sum_{c=1}^{C}\left(\sum_{k=1}^{K}\frac{E{\left(s_{ck}-\mu \right)}^{4}}{\sigma^{4}}P(s_{ck})/\mathrm{var}(s_{c1},\ldots ,s_{cK})\right)$$

where $\left\{s_{c1},\ldots ,s_{cK}\right\}$ is the band divided by $\left\{b_{1},\ldots ,b_{K+1}\right\}$, E is the mathematical expectation, σ the standard deviation of the multivariate spectrum, P is the power spectrum, and var is the variance of the bandwidth $\left\{s_{c1},\ldots ,s_{cK}\right\}$.

*Note: The purpose of Equation (11) is to obtain K + 1 spectral lines, which divide the C-dimensional multi-spectrum into *C×K* spectral segments. To achieve this, we first use the FTC algorithm to identify potential spectral intervals. The spectral intervals selected by the FTC algorithm inherently exhibit energy concentration characteristics, as detailed in the literature* [26,27]*. However, the FTC algorithm is only applicable to single-channel signals, resulting in different spectral line positions across multiple channels. Therefore, we designed the optimization approach described in Equation (11).*

We treat the joint segmentation problem as selecting ***K*** elements from the set F as an ordered combination (in ascending order). We use the branch and bound method to select the optimal ***K*** shared spectral lines from the set F of unimodal intervals in the multivariate spectrum, aiming to maximize the objective function. The specific algorithm steps are as follows:

***Step 1:*** We take the boundaries of the unimodal intervals as the candidate spectral line set F, and sort F in ascending order. We set the number of shared spectral lines as ***K***, the upper bound for the candidate solution is $H_{1}=-\infty$, and the number of random sampling times ***M***. The set of shared spectral lines is denoted as L=[].

***Step 2:*** Select candidate spectral lines $b_{i}$ from the set F (where $b_{i}>\max(L)$).

***Step 3:*** Randomly select the remaining spectral lines $b_{i+1},\ldots ,b_{K}$ from the set F, and compute the objective function J using Equation (11).

***Step 4:*** Return to ***Step 3*** and repeat the process until the maximum number of iterations ***M*** is reached. Set $H_{2}$ for the candidate spectral lines $b_{i}$ as maximum objective function value $J_{\max}$ obtained from the random selecting.

***Step 5:*** If $H_{2}>H_{1}$, update $H_{1}=H_{2}$.

***Step 6:*** Return to ***Step 2*** and repeat the process until all candidate spectral lines have been traversed. The spectral lines corresponding to $H_{1}$ are denoted as $b_{H_{1}}$.

***Step 7:*** Update $L=[L,b_{H_{1}}]$ and return to ***Step 2***, repeating the process until ***K*** shared spectral lines are obtained.

### 2.3. MBMD for Gear Fault Diagnosis

Gear fault signals often show energy concentration in specific frequency ranges and exhibit strong quasi-periodic characteristics, typically arising from uneven wear or other faults. These signals often feature prominent sidebands around the meshing frequency. Current decomposition methods do not fully account for these unique features of gear fault signals, hindering precise feature extraction.

Drawing inspiration from the gear fault mechanism, this paper presents a new multivariate signal decomposition method, MBMD. Initially, MBMD treats multivariate vibration signals as responses from a gear system in different operating states. By employing SAFD, the mechanical system is inverted, and the multivariate vibration signals are unified into a single Blaschke product, forming a Blaschke multi-spectrum that facilitates multi-channel information fusion. Next, MBMD applies a joint spectral segmentation strategy to partition the multivariate spectrum into energy-concentrated segments, thereby achieving modal alignment. Finally, MBMD constructs a voting filter bank that exploits the sideband characteristics of fault signals, effectively reducing noise interference while preserving the sideband features. The algorithm steps for MBMD are as follows:

***Step 1:*** Using multivariate vibration signals $\left\{f_{1},f_{2},\ldots ,f_{C}\right\}$ as the observational response of the gear system, the system is inverted using SAFD to obtain the common Blaschke product $\left\{B_{1},B_{2},\ldots ,B_{n}\right\}$ and construct the Blaschke multi-spectra $\left\{S_{1},S_{2},\ldots ,S_{C}\right\}$.

***Step 2:*** Divide $\left\{S_{1},S_{2},\ldots ,S_{C}\right\}$ into several unequal unimodal intervals and denote the set of unimodal interval boundaries as F.

***Step 3:*** Use the branch-and-bound method to find the optimal ***K + 1*** shared spectral lines in the set L, denoted as $\left\{b_{1},\ldots ,b_{K},b_{K+1}\right\}$.

***Step 4:*** Use the shared spectral lines to segment the Blaschke multi-spectra, resulting in C×K spectral segments, as shown in Equation (12).

(12) $$\left\{\begin{array}{c} S_{1}=\{s_{11},s_{12},\ldots ,s_{1K}\}\\ \vdots \\ S_{C}=\{s_{C1},s_{C2},\ldots ,s_{CK}\} \end{array}\right.$$

where $\left\{s_{c},s_{c2},\ldots ,s_{cK}\right\}$ represents the ***K*** spectral segments obtained by dividing $S_{i}$ according to $\left\{b_{1},\ldots ,b_{K},b_{K+1}\right\}$, and ∀c∈[1,C].

***Step 5:*** Employing Equation (13), perform the inverse ISAFD transform on the C×K spectral segments to obtain C×K intrinsic mode components, as shown in Equation (14).

(13) $$IMF_{ck}=ISAFD(S_{c})=\sum_{n=b_{k}}^{b_{k+1}}S_{c}(n)B_{n}$$

where ∀c∈[1,C], and ∀k∈[1,K].

(14) $$IMF=\left(\begin{array}{ccc} IMF_{11} & \ldots & IMF_{1K}\\ \vdots & \ddots & \vdots \\ IMF_{C1} & \ldots & IMF_{CK} \end{array}\right)$$

***Step 6:*** Perform frequency analysis on $IMF_{ck}$, and denote the corresponding frequency spectrum as $F_{ck}$. Select the top H spectral lines with the highest energy intensity as potential main frequencies (gear meshing frequency and its harmonics).

***Step 7:*** Select any potential main frequency, denoted as $m_{h}$, ∀h∈[1,H]. Based on Equation (15), iteratively calculate the sideband voting value, denoted as $V_{h}$.

(15) $$V_{h}(i)=F_{ck}(m_{h})\times (\sum_{g=1}^{G_{1}}F_{ck}(m_{h}-g\times i)+\sum_{g=1}^{G_{2}}F_{ck}(m_{h}+g\times i))$$

where i represents the position of the gear sideband spacing, $G_{1}$ and $G_{2}$ are the sideband spacing voting ranges, and their estimation methods are detailed in the Appendix of [28].

***Step 8:*** Return to ***Step 7*** and repeat until all possible main frequencies $\left\{m_{1},m_{2},\ldots ,m_{h}\right\}$ have been traversed. denote all sideband voting value as $V=\{V_{1},V_{2},\ldots ,V_{H}\}$.

***Step 9:*** Calculate the max value of $V=\{V_{1},V_{2},\ldots ,V_{H}\}$, and denoted the corresponding main frequency and sideband spacing as f and I.

***Step 10:*** Reconstruct the frequency spectrum according to Equation (16), remove the noise components, and convert the reconstructed frequency spectrum to the time domain using Equation (17), which $dIMF_{ck}$ is the final denoised result.

(16) $$F_{ck}(j)=\left\{\begin{array}{ll} F_{ck}(j) & \mathrm{if}\;j=f+i\times I\\ 0 & \;\mathrm{otherwise}. \end{array}\right.$$

where i is integer and $i\in [-G_{1},G_{2}]$.

(17) $$dIMF_{ck}=FFT^{-1}(F)$$

where $F\;F\;T^{-1}$ is inverse Fourier transform.

***Step 11:*** Return to ***Step 6*** until traversing all IMF, the final decomposition result is shown in Equation (18).

(18) $$dIMF=\left(\begin{array}{ccc} dIMF_{11} & \ldots & dIMF_{1K}\\ \vdots & \ddots & \vdots \\ dIMF_{C1} & \ldots & dIMF_{CK} \end{array}\right)$$

**Figure 1:**
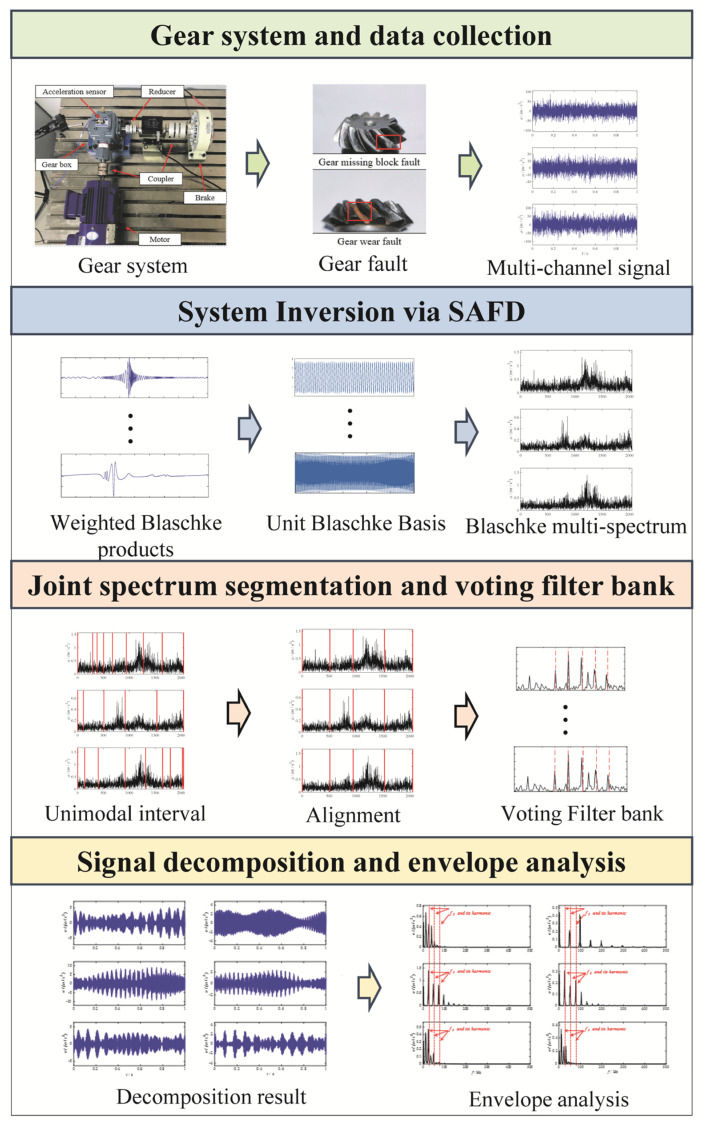
The flowchart of MBMD.

In summary, the flowchart of gear fault diagnosis based on MBMD algorithm is shown in Figure 1.

## 3. Gear Vibration Signal Experiment

**Figure 2:**
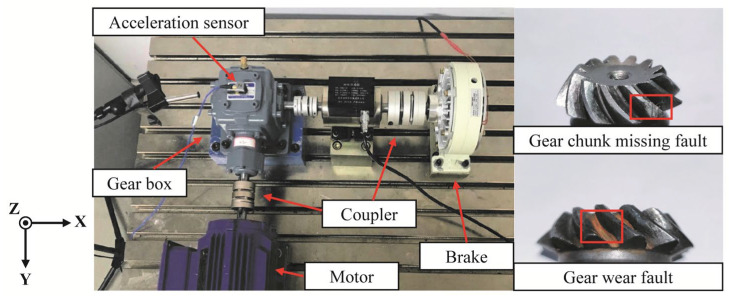
Gear vibration signal experiment platform.

To assess the performance of MBMD in mechanical transmission systems, we conducted two types of gear fault experiments. The test rig used in the experiments consisted of a bevel gear setup, which included a DC motor, couplings, a brake, bearings, and a pair of grease-lubricated bevel gears, as depicted in Figure 2. The driving gear has 12 teeth, while the driven gear has 24 teeth, resulting in a transmission ratio of 2. Vibration acceleration signals were captured using the LMS SCM09 data acquisition system, manufactured by Siemens, Germany, and then transmitted to a computer for further processing.

### 3.1. Gear Chunk Missing Fault Experiment

**Figure 3:**
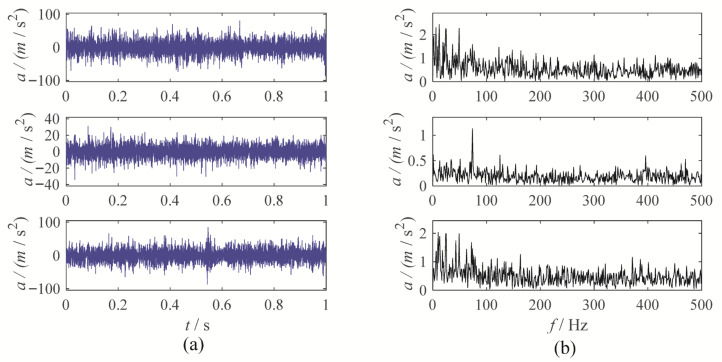
The waveforms and envelope spectra of multi-channels gear chunk missing signal: (**a**) waveform; (**b**) Envelope spectra.

A gear chunk missing multi-channel fault diagnosis experiment is designed to evaluate the decomposition performance of MBMD. In this experiment, the motor speed is set to 1500 RPM, with the fault characteristic frequency fixed at 25 Hz. Vibration data is collected simultaneously from three channels—X, Y, and Z—using accelerometers. Figure 3 illustrates the time-domain waveforms of the vibration signals from these three channels, along with their corresponding envelope spectra.

As shown in Figure 3, the fault characteristic frequency is masked by noise, which complicates the assessment of the condition. To diagnose the gearbox system, MBMD is applied to decompose the muti-channel signal. The decomposition parameters for MBMD are chosen based on prior experience, with the number of decomposition modes set to 4 and the smoothing filter window size set to 50.

**Figure 4:**
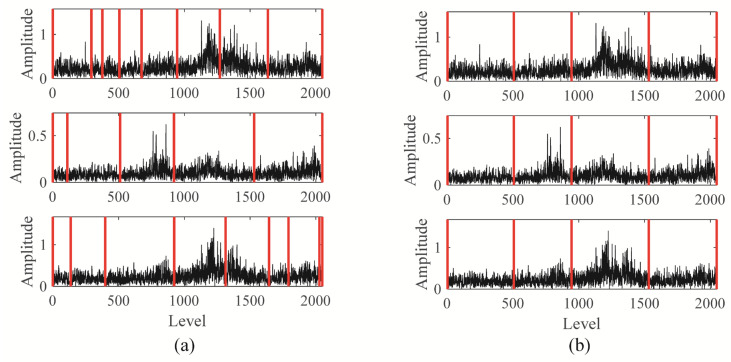
The Blaschke muti-spectra and segmentation result: (**a**) result of unimodal segments; (**b**) result of joint spectrum segments.

The Blaschke multi-spectrum and segmentation result is shown in Figure 4 (Each sub-figure in Figure 4 corresponds to the Blaschke multi-spectrum of the signals shown in Figure 3). Upon examination, it is evident that the multi-spectrum is divided into four distinct regions by the shared spectral lines, with each region demonstrating varying levels of energy concentration. Upon observation, we can see that the Blaschke spectrum of the multivariate signal is divided into four intervals by the common spectral lines, with each interval showing an energy concentration trend. Among these, the third spectral interval of the first and third channels, as well as the second spectral interval of the second channel, exhibit the most pronounced energy concentration trend. The regions with the most evident energy concentration trend also correspond to the spectral areas associated with the gear fault modes.

**Figure 5:**
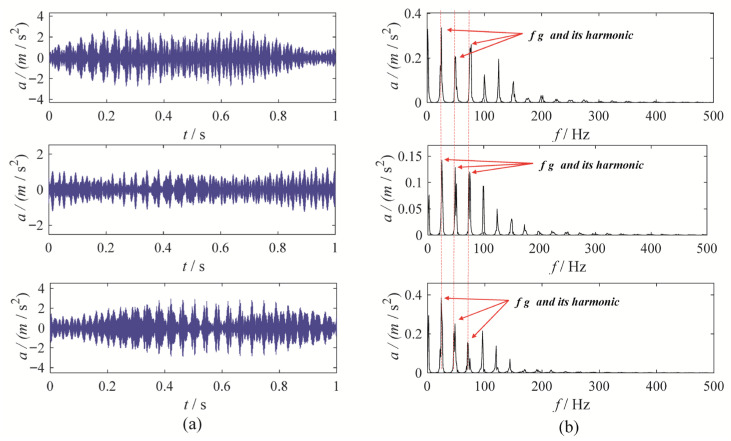
The MBMD results: (**a**) time-domain waveform; (**b**) envelope spectrum.

MBMD decomposes the signal from each channel into four modes. The mode exhibiting the highest correlated kurtosis is chosen as the optimal mode for each channel. Specifically, the third mode derived from the data of the first and third channels, and the second mode from the second channel, are chosen as the optimal modes. Figure 5 displays the waveform and envelope spectrum for these optimal modes.

Upon examination, the time-domain waveform obtained from the MBMD clearly exhibits distinct amplitude modulation and frequency modulation patterns. The optimal mode identified by MBMD displays a strong amplitude at the fault characteristic frequency and its harmonics, with minimal surrounding interference, indicating a fault in the gearbox system.

**Figure 6:**
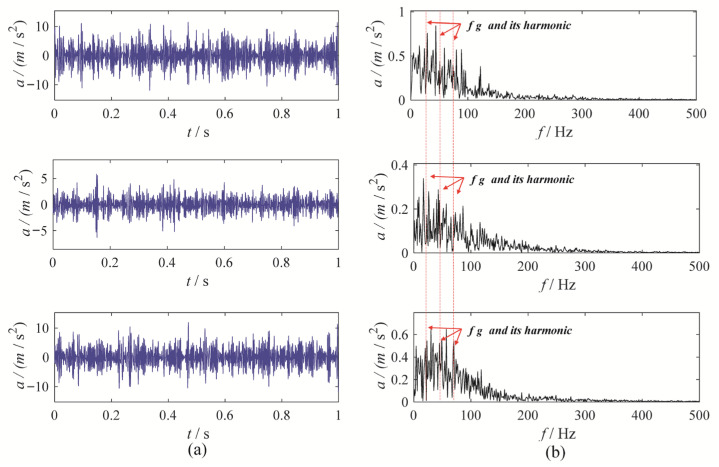
The MEMD results: (**a**) time-domain waveform; (**b**) envelope spectrum.

**Figure 7:**
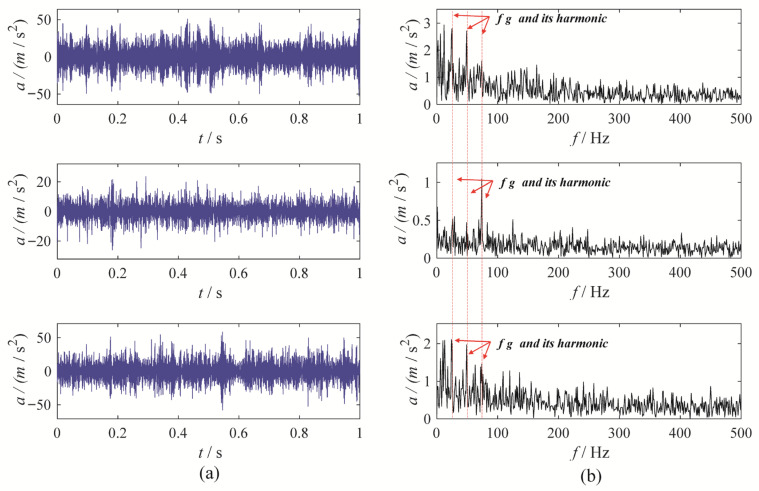
The CAPMLCD results: (**a**) time-domain waveform; (**b**) envelope spectrum.

**Figure 8:**
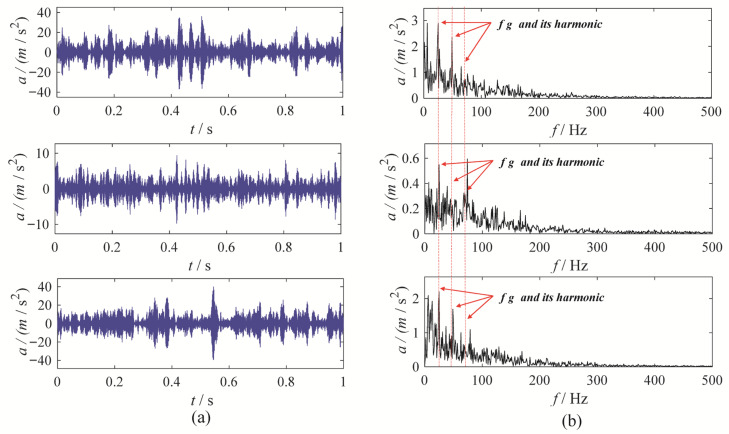
The MVMD results: (**a**) time-domain waveform; (**b**) envelope spectrum.

**Figure 9:**
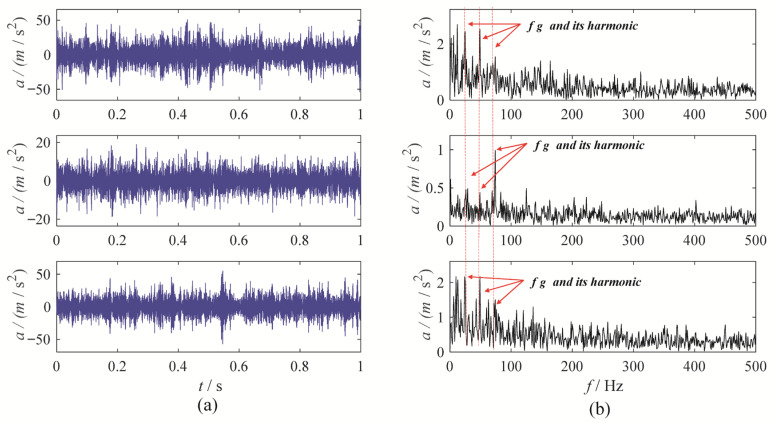
The MLCD results: (**a**) time-domain waveform; (**b**) envelope spectrum.

**Figure 10:**
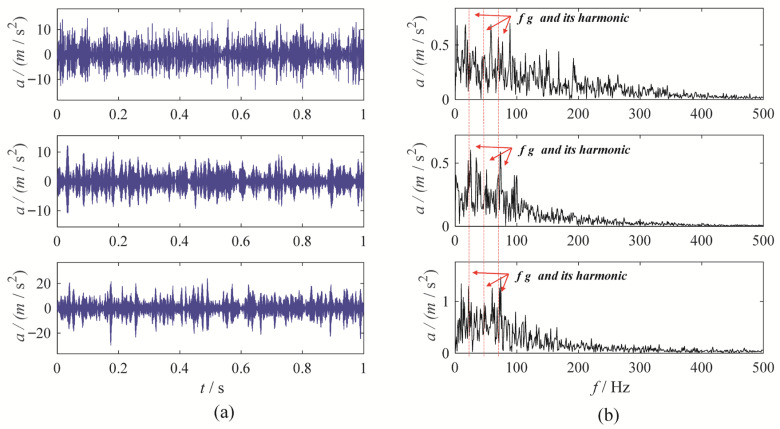
The MCMMD results: (**a**) time-domain waveform; (**b**) envelope spectrum.

To further validate the effectiveness of MBMD, we applied five other methods—CAPMLCD, MEMD, MVMD, MLCD and MCMMD [8]—to the same signal. The decomposition parameters for these methods were selected based on empirical considerations. For MEMD, MLCD and CAPMLCD, the projection numbers were set to 64, while for MVMD and MCMMD, the penalty factor was set to 3000, the number of modes was set to 5, and the convergence criterion was set to 1 × 10^−6^. The optimal mode was selected based on the correlated kurtosis, and the decomposition outcomes of the three comparison methods are presented in Figure 6, Figure 7, Figure 8, Figure 9 and Figure 10. Among them, the first mode derived from decomposing the three-channel data using CAPMLCD, MLCD and MEMD is regarded as the best mode, whereas the fifth mode from the MVMD and MCMMD across the three channels is regarded as the optimal one.

Upon observation, the decomposition results from MEMD, MLCD, MCMMD, and CAPMLCD fail to identify the fault characteristic frequency in the envelope spectrum, making the evaluation of the gearbox system’s condition more challenging. In contrast, MVMD reveals a clear amplitude at both the fault characteristic frequency and its harmonics in the envelope spectrum of the fifth mode obtained from the three-channel data decomposition. However, its performance is inferior to that of MBMD. Therefore, MBMD outperforms the other methods in terms of decomposition ability, effectively extracting gear fault characteristics.

### 3.2. Gear Wear Experiment

**Figure 11:**
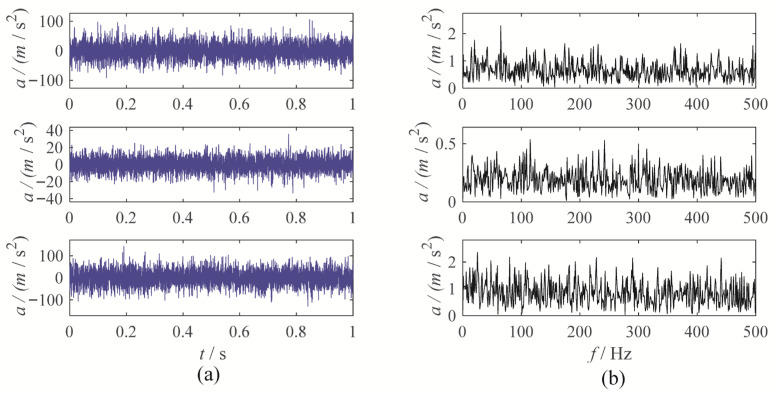
The waveforms and envelope spectra of multi-channels gear wearing signal: (**a**) waveform; (**b**) Envelope spectrum.

To further evaluate the performance of MBMD, a multi-channel wear fault diagnosis experiment is conducted to test its decomposition capabilities. In this experiment, the motor runs at a speed of 1500 rpm, and the fault characteristic frequency is set at 25 Hz. Vibration data is captured from three channels—X, Y, and Z—using an accelerometer. Figure 11 illustrates the waveforms and their corresponding envelope spectra for the vibration signals from the three channels.

**Figure 12:**
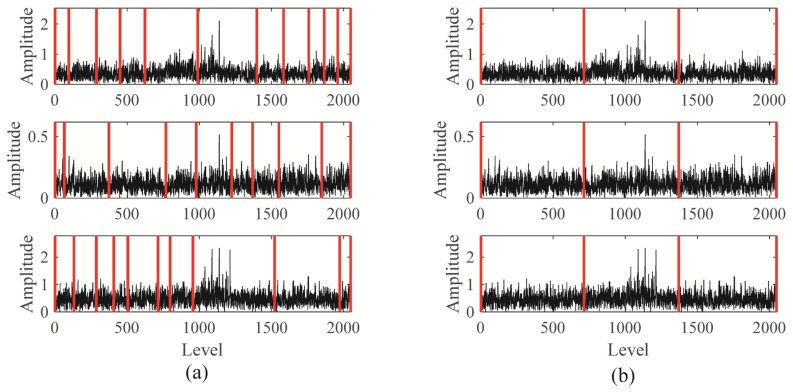
The Blaschke muti-spectra segmentation: (**a**) Unimodal segments; (**b**) Joint spectrum segments.

To accurately diagnose faults, MBMD is applied to decompose the muti-channel signal. The joint segmentation diagram of MBMD is shown in Figure 12. Upon examination, it is evident that the Blaschke multi-spectra is partitioned into three regions by the shared spectral lines. By observation, we can see that the Blaschke spectrum of the multivariate signal is divided into three intervals, each exhibiting an energy concentration trend. Among these, the second spectral interval of the multivariate signal shows the most concentrated energy. The regions with the most evident energy concentration trend also correspond to the spectral areas associated with the gear fault modes.

**Figure 13:**
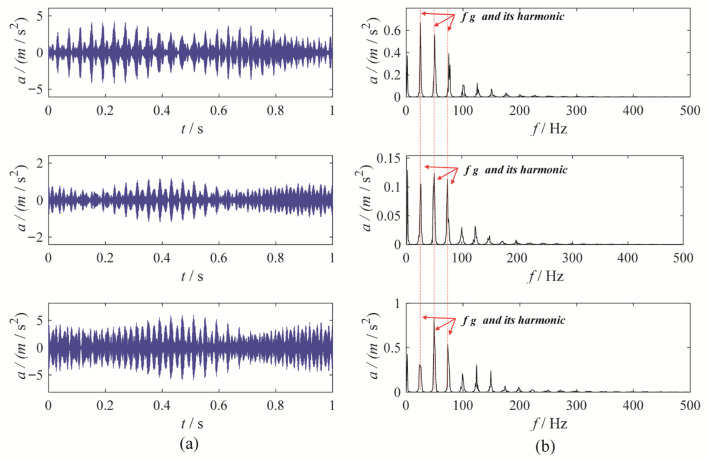
The MBMD results: (**a**) time-domain waveform; (**b**) envelope spectrum.

MBMD decomposes each channel’s signal into three modes. The mode exhibiting the highest correlated kurtosis is chosen as the optimal mode for every channel. Among these, the second mode obtained from the signal of three channels is selected as the optimal mode. The waveform and envelope spectrum of best mode are presented in Figure 13.

**Figure 14:**
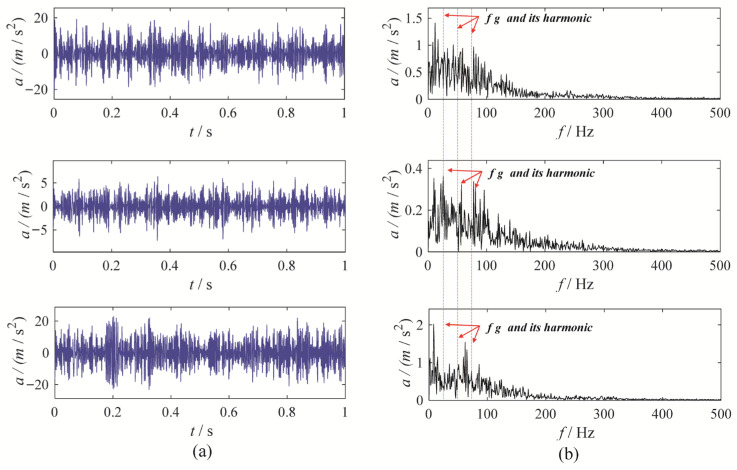
The MEMD results: (**a**) time-domain waveform; (**b**) envelope spectrum.

**Figure 15:**
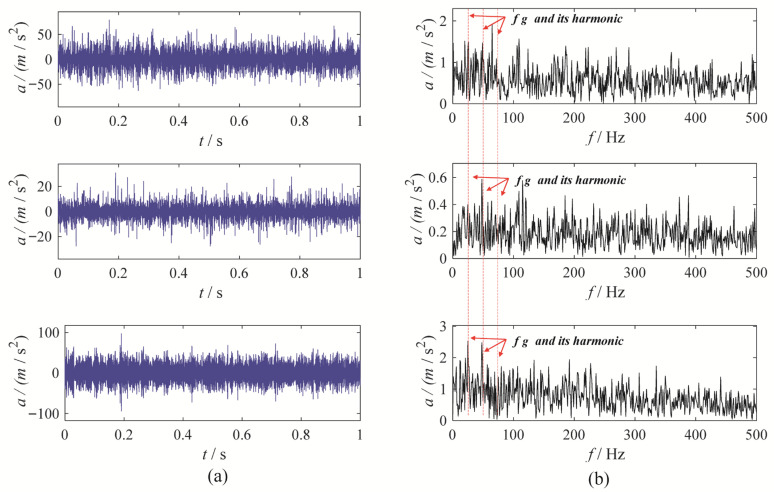
The CAPMLCD results: (**a**) time-domain waveform; (**b**) envelope spectrum.

**Figure 16:**
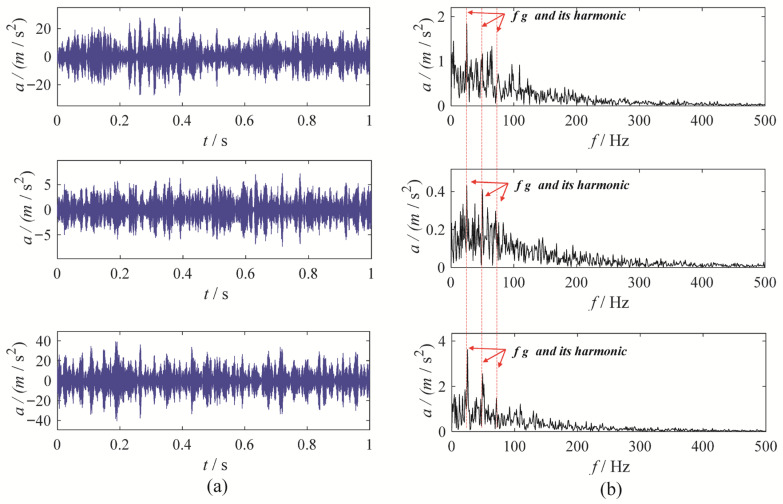
The MVMD results: (**a**) time-domain waveform; (**b**) envelope spectrum.

**Figure 17:**
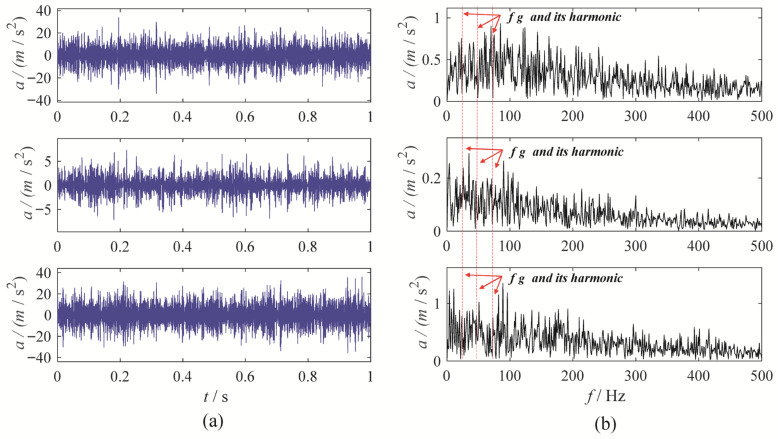
The MLCD results: (**a**) time-domain waveform; (**b**) envelope spectrum.

**Figure 18:**
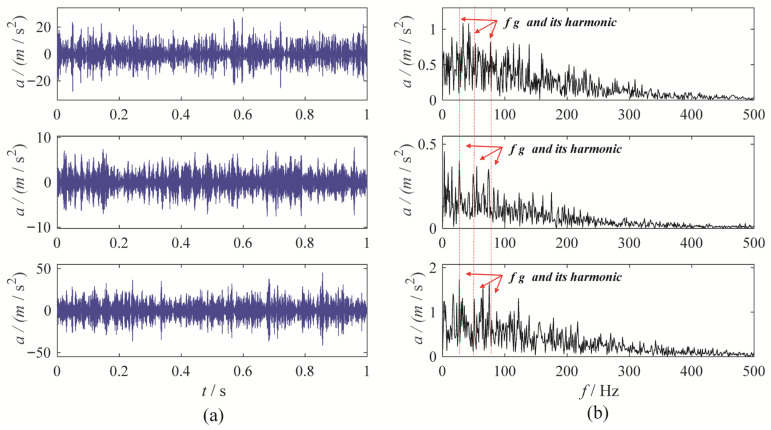
The MCMMD results: (**a**) time-domain waveform; (**b**) envelope spectrum.

The MBMD results reveal a distinct amplitude at the fault characteristic frequency and its harmonics, free from surrounding interference, enabling a clear identification of the fault in the gearbox system. To further demonstrate the effectiveness of MBMD, we applied five other multivariate signal decomposition methods—CAPMLCD, MEMD, MLCD, MCMMD [8] and MVMD—on the same multivariate signal from a gear wear fault. The decomposition results from these three methods are presented in Figure 14, Figure 15, Figure 16, Figure 17 and Figure 18.

Upon analysis, it becomes evident that the envelope spectra of the MEMD, MLCD, and MCMMD results do not reveal the gear fault characteristic frequency, which complicates the assessment of the gearbox system’s condition. The fourth component from the CAPMLCD of the second channel data shows a prominent amplitude at the fault characteristic frequency, though other components fail to highlight the fault features. In contrast, the envelope spectrum of the fifth mode obtained from the MVMD of the three-channel data exhibits significant amplitude at both the fault characteristic frequency and its harmonics, albeit with some residual noise interference. Overall, its performance is inferior to that of MBMD. This is primarily because MBMD incorporates mechanical system characteristics into the multivariate signal decomposition process, allowing it to more accurately capture gear fault features while minimizing noise interference.

Based on the analysis above, it is evident that MBMD surpasses the other methods in decomposition capability. It effectively extracts gear fault feature information and offers significant practical value.

## 4. Conclusions

In response to the issues with existing multivariate signal decomposition methods, which failed to fully consider the mechanical system characteristics and struggled to accurately extract gear fault features, this paper proposed a novel multivariate signal decomposition method, Multivariate Blaschke-based Mode Decomposition (MBMD). First, MBMD treated multivariate vibration signals as the observed responses of a mechanical system in different dimensions. Based on the theory of Stochastic Adaptive Fourier Decomposition (SAFD), it inverted the mechanical system and represented multivariate vibration signals using a unified Blaschke product, thereby achieving multi-channel information fusion. Next, MBMD constructed a Blaschke multi-spectrum, transforming the multivariate signal decomposition problem into a multivariate spectrum segmentation issue. A joint spectral segmentation algorithm was then proposed to partition the Blaschke multi-spectra into spectral segments with the same Blaschke product, achieving modal alignment. Finally, based on the gear fault mechanism, MBMD designed voting filter banks to eliminate noise interference in the modes and accurately extract fault feature information. As a novel multivariate signal decomposition method, MBMD dynamically integrated multivariate data and accurately extracted fault feature information. We demonstrated the effectiveness of MBMD in early gear fault detection through two cases: gearbox chunk missing fault and wear fault, providing a new perspective for mechanical vibration signal analysis.

Although MBMD performs excellently in handling multivariate vibration signals of mechanical systems, it has certain limitations because it only uses observed data to invert the rotating mechanical system. Blaschke products obtained from inversion may not accurately reflect the gear system characteristics when the system is complex or the observation data is limited, which can lead to deviations in the decomposition results. In future research, we plan to incorporate more mechanical mechanism information into the inversion process to achieve a more accurate reconstruction of the system state. Moreover, investigating an adaptive version of MBMD is also considered a promising research direction.

## Data Availability

The authors do not have permission to share data.
